# Safety of inter-hospital transfer of patients with acute ischemic stroke for evaluation of endovascular thrombectomy

**DOI:** 10.1038/s41598-020-62528-4

**Published:** 2020-03-27

**Authors:** Lars-Peder Pallesen, Simon Winzer, Kristian Barlinn, Alexandra Prakapenia, Timo Siepmann, Cosima Gruener, Johannes Gerber, Kevin Haedrich, Jennifer Linn, Jessica Barlinn, Volker Puetz

**Affiliations:** 1 Department of Neurology, Dresden Neurovascular Center, Carl Gustav Carus University Hospital, Technische Universität Dresden, Dresden, Germany; 2 Institute of Neuroradiology, Dresden Neurovascular Center, Carl Gustav Carus University Hospital, Technische Universität Dresden, Dresden, Germany

**Keywords:** Outcomes research, Stroke, Stroke

## Abstract

Stroke networks facilitate access to endovascular treatment (EVT) for patients with ischemic stroke due to large vessel occlusion. In this study we aimed to determine the safety of inter-hospital transfer and included all patients with acute ischemic stroke who were transferred within our stroke network for evaluation of EVT between 06/2016 and 12/2018. Data were derived from our prospective EVT database and transfer protocols. We analyzed major complications and medical interventions associated with inter-hospital transfer. Among 615 transferred patients, 377 patients (61.3%) were transferred within our telestroke network and had transfer protocols available (median age 76 years [interquartile range, IQR 17], 190 [50.4%] male, median baseline NIHSS score 17 [IQR 8], 246 [65.3%] drip-and-ship i.v.-thrombolysis). No patient suffered from cardio-respiratory failure or required emergency intubation or cardiopulmonary resuscitation during the transfer. Among 343 patients who were not intubated prior departure, 35 patients (10.2%) required medical interventions during the transfer. The performance of medical interventions was associated with a lower EVT rate and higher mortality at three months. In conclusion, the transfer of acute stroke patients for evaluation of EVT was not associated with major complications and transfer-related medical interventions were required in a minority of patients.

## Introduction

Current evidence from randomized controlled trials supports endovascular therapy (EVT) for patients with large vessel occlusion (LVO)^1^. Whilst the procedure is now standard of care, it is hampered by its overall limited availability^2,3^. Among strategies to improve EVT availability for stroke patients are telemedical stroke networks which have been shown to effectively facilitate early initiation of treatment^4,5^. In these networks patients are admitted to the nearest community hospital for telemedicine based assessment of intravenous thrombolysis (IVT) followed by transfer to the stroke center if eligibility for EVT is determined (“drip-and-ship”)^6–9^. Depending on the geographical extent of stroke networks, patients may have to be transferred over long distances. During transfer, acute stroke patients can be exposed to complications and neurological worsening^10,11^. Therefore one might argue that a physician, preferably with experience in critically ill patients, should routinely accompany the transfer of stroke patients. However, requesting a physician and the limited availability of qualified personnel in smaller hospitals may result in delay of transport^9,11,12^. As the benefit from EVT is time dependent, one needs to outweigh the decreased likelihood to achieve a good neurological outcome if patient transfer is delayed significantly with the risk of medical complications during the transport. Whether patient transfer within telemedical stroke networks is safe and routinely requires an accompanying physician has not been studied systematically.

We therefore sought to analyze the risk of medical complications and the need for specific medical interventions during inter-hospital transfer of acute ischemic stroke patients with LVO who are under evaluation for EVT in a stroke network.

## Methods

### Study design and population

We performed a retrospective analysis of prospectively collected data of adult patients with an acute ischemic stroke who were evaluated for EVT at our center. In our ongoing EVT database detailed information on demographics, clinical status, vascular risk factors, imaging, stroke scores and treatment as well as modified Rankin scale (mRS) scores assessed at 90 days are prospectively recorded. For this study we analyzed all consecutive patients between 06/2016 to 12/2018 who were transferred to our center for assessment of EVT from remote telestroke hospitals after teleconsultation or from remote neurology departments without EVT available as part of our stroke network. We excluded patients who were directly admitted to our tertiary stroke center or who were transferred from remote hospitals outside our stroke network.

### The stroke network

**Figure 1:**
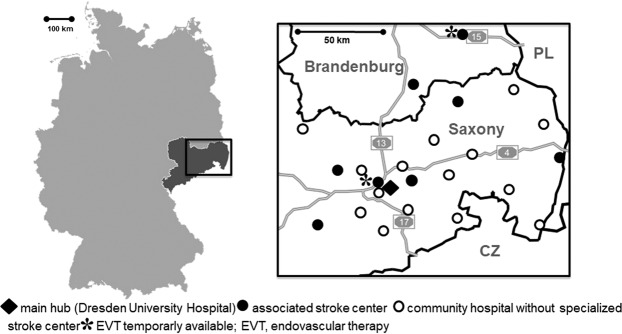
Left: Map of the Federal Republic of Germany with the state of Saxony highlighted in dark grey. Right: Map of eastern Saxony and southern Brandenburg with the participating hospitals of the Stroke Eastern Saxony Network (SOS-NET). Black lines indicate state and country borders; grey lines with numbers indicate highways (*Autobahn*). CZ, Czech Republic; PL, Poland.

The Stroke Eastern Saxony and Southern Brandenburg Network (SOS-NET) is a telestroke network covering the eastern part of the German state of Saxony and the southern part of the German State of Brandenburg with a catchment area of approximately 2.4 million people (Fig. 1)^13^. During the observation period, the network provided telestroke expertise for 15 smaller community hospitals without neurology departments or certified stroke units^13^. Furthermore, eight associated hospitals with neurology departments and certified stroke-unit were part of the network. Of these, two offered EVT during the study period, but not on 24/7 basis.

The decision for IVT and transfer to the main hub for evaluation of EVT is either made by the stroke fellow based on the patients´ medical history, telemedical image assessment and clinical examination, or by the neurologist at the hospitals with on-site stroke unit but without EVT available^13,14^.

After arrival at our center, we routinely perform repeated imaging with CT and CTA and additional multiphase CTA and/or perfusion-CT in unclear scenarios (e.g., late time-window) to determine final EVT-eligibility per institutional criteria. Final decision for EVT is made by the stroke-neurologist and interventional neuroradiologist on-call.

The study has been approved by the institutional research ethics committee of the Technische Universität Dresden (#272072017) and has therefore been performed in accordance with the ethical standards laid down in the 1964 Declaration of Helsinki and its later amendments. Since we used observational data from an ongoing registry informed formal consent was waived according to local legislation; however, patients or their legally authorized representatives gave approval for treatment with intravenous thrombolysis and/or EVT, where possible.

### Patient transfer within the telestroke network

The transfer of patients in our telestroke network is provided by ambulance or by helicopter based on current availability and distance. The decision about the transfer modality is made by the transferring hospital and the emergency-physician coordinating center. Per current protocol all patients are routinely accompanied by an emergency physician or by physicians from the remote hospital. For helicopter transport the patient is always accompanied by an emergency-physician as part of the helicopter crew.

For analysis of the transfer, we retrospectively evaluated the electronically stored transfer protocols. These transfer protocols are provided by the medical staff of the transporting unit after arrival of the patient and contain a synopsis of the patients´ clinical history and clinical course as well as information regarding blood pressure, heart rate, oxygen saturation and Glasgow Coma Scale (GCS) scores prior to departure at the transferring hospital and on arrival at our center. Furthermore, all administered medication and complications are documented.

### Study endpoints

As the primary endpoint we used any major medical complication that occurred during the transfer defined as emergency intubation, cardiopulmonary resuscitation or death of the patient during transfer. Secondary endpoints were medical interventions defined as the requirement for intravenous medication (e.g., antihypertensive drugs, anti-vomiting medication, sedatives etc.); and neurological worsening defined as an increase of the NIHSS score by >4 points between departure and arrival. We also assessed neurological improvement during transfer defined as a decrease of the NIHSS score by >4 points between departure and arrival.

### Statistical analysis

Statistical analyses were performed with SciPy 1.2.1, Pandas 0.24.2, Statsmodels 0.10.0 with Python 3.7.3. (University of Michigan, Ann Arbor, MI, United States). Continuous and non-continuous variables are presented as median [interquartile range, IQR] and percentage. Statistical comparisons were performed using Chi-square test, Fisher’s exact test, t-test and Mann-Whitney-U-test, where appropriate. Significant results from the univariate analysis were tested using a multivariate logistic regression analysis. Also age as a clinical characteristic deemed relevant *a priori* was included in the multivariate model although it was not significant in the univariate analysis. We considered a p-value < 0.05 as statistically significant for all analyses.

## Results

### Patient population

**Figure 2:**
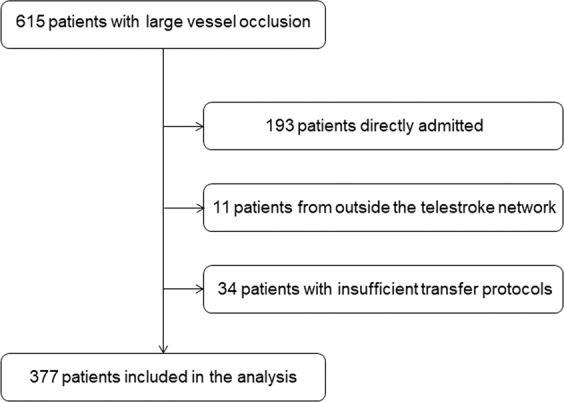
Study flow diagram.

In the observed time period, 615 patients were admitted for evaluation of EVT to our stroke center of whom 422 patients (68.6%) were transferred from remote hospitals. Of these, we excluded 11 patients (2.6%) as they were transferred from nearby community hospitals outside the stroke network. Further 34 patients (8.1%) were excluded due to insufficient or missing transfer protocols, leaving 377 patients for the final analysis (Fig. 2). Of these, 137 patients (36.3%) were transported via helicopter and 240 (63.7%) patients via ambulance. The overall median age was 76 years (IQR 17) and 190 (50.4%) patients were male. Further baseline characteristics are summarized in Table 1.

**Table 1:** Baseline characteristics of the study population; EVT indicates endovascular therapy; IQR, interquartile range; NIHSS, National Institute of Health Stroke Scale; mRS, modified Rankin Scale; mmHg, millimeter mercury.

Number, n	377
Age, years, median (IQR)	76.0 (17)
Male gender, % (n/N)	50.4 (190/377)
Intravenous thrombolysis, % (n/N)	65.3 (246/377)
EVT performed, % (n/N)	60.7 (229/377)
Vascular risk factors	
Diabetes mellitus, % (n/N)	29.9 (112/375)
Atrial fibrillation, % (n/N)	53.9 (202/375)
Pre-existing stroke, % (n/N)	14.9 (56/375)
Arterial hypertension, % (n/N)	84.8 (318/375)
Hyperlipidemia, % (n/N)	27.4 (64/234)
Current smoking, % (n/N)	6.4 (24/377)
Coronary artery disease, % (n/N)	5.7 (12/212)
Basilar artery occlusion, % (n/N)	9.5 (36/377)
Transfer by helicopter, % (n/N)	36.3 (137/377)
Intubation prior transfer, % (n/N)	9 (34/377)
Transfer time, minutes, median (IQR)	35.0 (18)
NIHSS on departure, median, (IQR)	17.0 (8.0)
Systolic blood pressure on departure, mmHg, median (IQR)	150.0 (35.0)
Heart rate on departure, bpm, median (IQR)	78.0 (21.0)
Oxygen saturation on departure, percent, median (IQR)	97.0 (3.0)
Glascow Coma Scale on departure, median (IQR)	13.0 (5.0)

The median transfer time (door-to-door time) was 34 min (IQR 14) with helicopter-transfer and 36 min (IQR 21.3) with ambulance-transfer. Of the 161 patients who received IVT in a drip-and-ship approach, 55 (34.2%) patients were transported by helicopter and 106 (65.8%) patients by ambulance. Thirty-four (9.0%) of all patients were intubated prior to transfer with a significantly higher rate in patients with basilar artery occlusion (BAO) compared to patients with anterior circulation stroke (12 of 28 patients [42.9%] vs. 22 of 349 patients [6.3%]; Odds ratio [OR] 11.15; 95%CI 4.70–26.45).

After arrival and repeated imaging at our center, 229 of the 377 patients (60.7%) were eligible for EVT and underwent the procedure. Reasons not to perform EVT were extensive ischemic changes on CT characterized by an ASPECTS score <5 in 75 patients (50.7%), vessel recanalization in 55 patients (37.2%), absence of a CTP-based mismatch in a time-window>6 hours in 8 patients (5.4%), mild neurological deficits characterized by an NIHSS score <5 points in 7 patients (4.7%), pre-existing comorbidities unknown prior to transfer in 2 patients (1.3%) and expected major technical difficulties to perform angiography due to extensive vessel tortuosity in 1 patient (0.7%).

At three months, 108 of all 377 patients (28.6%) had an independent functional outcome (mRS 0–2) and 118 patients (31.3%) were deceased. In comparison, 75 of 193 (38.9%) of the directly admitted patients were deceased after three months (p = 0.08). The performance of EVT was associated with a higher likelihood for an independent functional outcome (mRS scores 0–2: 73 [31.8%] patients vs. 35 [23.6%] patients) and reduced mortality (62 [27.1%] patients vs. 56 [37.8%] patients).

### Primary and secondary outcome parameters

Regarding our primary outcome parameter, none of the transferred patients required cardiopulmonary resuscitation or emergency intubation during transfer. Furthermore, no patient died during transfer. The comparison of clinical and cardiorespiratory parameters between departure in the remote hospital and on arrival at our center showed no clinically relevant differences (Table 2).

**Table 2:** Vital signs and clinical parameters on departure at the telestroke hospital and on arrival at the main hub of the whole study population. Bpm indicates beats per minute; NIHSS, National Institute of Health Stroke Scale; IQR, interquartile range; mmHg, millimeter mercury.

Clinical parameters/Vital signs	At departure	On arrival	p
NIHSS, median (IQR)	17 (8)	17 (9)	0.23
Systolic Blood pressure, mmHg, median (IQR)	150 (35)	150 (34)	0.41
Heart rate, bpm, median (IQR)	78 (21)	79 (23)	0.39
Oxygen saturation, percent, median (IQR)	97 (3)	97 (3)	0.20
Glasgow Coma Scale, median (IQR)	13 (5)	13 (5)	0.47

In the group of 343 patients who were not intubated prior to transfer, any intravenous medication during the transfer was applied in 35 patients (10.2%). Reasons to apply medication are summarized in Table 3. The majority of medical interventions was required for elevated blood-pressure. Medical interventions were more frequently required in patients who received intravenous thrombolysis prior to transfer in a drip-and-ship approach (29 of 35 [82.9%] patients vs. 192 of 308 [62.3%] patients; p = 0.016; Table 4). In contrast, only one (6.3%) of the non-intubated patients with BAO required medication during transfer.

**Table 3:** Complications and given medication during transfer. Note that the number of administered drugs exceeds the number of total patients with medical intervention (n = 35) due to cases in which more than one drug was given.

Complication	Medication
High blood pressure	Urapidil (n = 22), Nitrendipine (n = 1), Nitroglycerine (n = 1), Metoprolol (n = 2)
Nausea	Dimenhydrinate (n = 3), Pantoprazole (n = 1), Ondansetron (n = 1), Metoclopramide (n = 2)
Agitation	Midazolam (n = 2), Lorazepam (n = 1)
Hypoglycemia	Intravenous glucose (n = 1)
Sinus bradycardia	Atropine (n = 1)
Low blood pressure	Theodrenalin-Cafedrin (n = 1)

**Table 4:** Comparison of non-intubated patients with and without medical intervention during transfer; bpm indicates beats per minute; EVT, endovascular therapy; IQR, interquartile range; NIHSS, National Insitute of Health Stroke Scale; mmHg, millimeter mercury; mRS, modified Rankin Scale.

	No intervention during transfer	Intervention during transfer	p
Age, years, median (IQR)	76.0 (17.0)	78.0 (13.5)	0.257
Sex, male, % (n/N)	49.7 (153/308)	40.0 (14/35)	0.365
Intravenous thrombolysis, % (n/N)	62.3 (192/308)	82.9 (29/35)	0.016
EVT performed, % (n/N)	64.0 197/308	40.0 (14/35)	0.009
Vascular risk factors			
Diabetes, % (n/N)	28.1 (86/306)	37.1 (13/35)	0.358
Atrial fibrillation, % (n/N)	53.6 (164/306)	54.3 (19/35)	0.919
Pre-existing stroke, % (n/N)	15.4 (47/306)	8.6 (3/35)	0.410
Arterial hypertension, % (n/N)	84.6 (259/306)	88.6 (31/35)	0.713
Hyperlipidemia, % (n/N)	30.3 (56/185)	22.2 (6/27)	0.527
Current smoking, % (n/N)	7.5 (23/308)	0.0 (0/35)	0.188
Coronary artery disease, % (n/N)	5.9 (11/185)	3.7 (1/27)	0.980
Transfer by helicopter, % (n/N)	32.1 (99/308)	48.6 (17/35)	0.079
Transfer time, minutes, median (IQR)	34.0 (16.0)	37.0 (23.5)	0.299
Three months mRS score 0–2, % (n/N)	30.9 (94/304)	26.5 (9/34)	0.694
Three months mRS score 6, % (n/N)	26.6 (81/304)	47.1 (16/34)	0.028
NIHSS on departure, median (IQR)	16.0 (7.0)	16.0 (5.5)	0.191
NIHSS at arrival, median (IQR)	16.0 (9.0)	16.0 (9.0)	0.208
Systolic blood pressure on departure, mmHg, median (IQR)	154.0 (33.0)	166.5 (37.25)	0.005
Systolic blood pressure at arrival, mmHg, median (IQR)	153.0 (31.0)	154.5 (29.25)	0.683
Heart rate on departure, bpm, median (IQR)	78.0 (20.0)	86.5 (32.0)	0.039
Heart rate at arrival, bpm, median (IQR)	79.0 (21.0)	87.0 (27.25)	0.113
Oxygen saturation on departure, percent, median (IQR)	97.0 (3.0)	95.0 (2.5)	0.001
Oxygen saturation at arrival, percent, median (IQR)	97.0 (3.0)	96.0 (2.5)	0.428
Glasgow Coma Scale on departure, median (IQR)	13.0 (4.0)	13.0 (5.0)	0.362
Glasgow Coma Scale at arrival, median (IQR)	13.0 (4.0)	12.0 (5.0)	0.181

Patients who required medical interventions during the transfer had a higher systolic blood pressure, a higher heart rate and a lower oxygen saturation on departure. The need for transfer-related medical interventions was associated with a lower EVT-rate (OR 0.38; 95%CI 0.18–0.77) and an increased mortality at 3-months (OR 2.36; 95%CI 1.16–4.81). In multivariate analysis, intravenous thrombolysis (OR 4.44; 95%CI 1.46–13.51), higher systolic blood pressure at departure (OR 1.02; 95%CI 1.01–1.04), higher heart rate at departure (OR 1.02; 95%CI 1.00–1.04) and lower oxygen saturation at departure (OR 0.91; 95%CI 0.88–0.94) were significantly associated with medical interventions during transfer.

When excluding patients who were intubated prior to transfer, 38 of 343 patients (11.1%) suffered a neurological worsening by>4 points on the NIHSS score during the transfer, of whom 24 patients (63.2%) were treated with IVT. In contrast, 64 patients (18.7%) improved clinically by >4 points on the NIHSS score during the transfer. Of these, 48 patients (75%) had received IVT of whom 23 patients (47.9%) demonstrated vessel recanalization on repeated CTA after arrival. Pre-existing coronary artery disease emerged as the sole predictor for relevant neurological decline during transfer (OR 5.26; 95%CI 1.63–16.99), while the transfer time itself was not associated with a neurological decline (OR 1.00; 95%CI 0.98–1.02).

## Discussion

In our study population, patients with acute ischemic stroke who were transferred for potential EVT in our stroke network did not suffer major complications during the transfer. Hence, no patient required cardiopulmonary resuscitation or emergency endotracheal intubation and no patient died. When excluding those patients who were intubated prior to transfer, any medical intervention was performed in 10.2% of all patients during transfer and these medical interventions were minor (e.g., application of antihypertensive medication). However, the requirement for transfer-related medical interventions was associated with a lower EVT rate and higher mortality at three months. Moreover, a significant percentage of patients (11.1%) experienced a significant clinical worsening during the transfer.

Since the publication of the randomized controlled trials to prove efficacy of interventional recanalization in patients with acute ischemic stroke due to LVO, the need to facilitate access to this treatment option for eligible patients is of growing scientific interest^15–19^. Collaborative stroke and telestroke networks can increase the number of patients with LVO who are evaluated for EVT^4,5^. However, this is at the cost of significant treatment delay due to initial evaluation in a remote hospital and subsequent secondary transfer, often preventing EVT in a large proportion of patients^11,16,20,21^. Nevertheless, considering the necessary facilities, expertise and maintenance costs linked to EVT, a ubiquitous distribution of EVT capacity seems to be unrealistic especially in rural areas^5^.

Our data support the general safety of patient transfer within telemedical stroke networks as part of a “drip-and-ship” strategy. Although we observed an association between transfer-related medical interventions with worse functional outcomes, none of our patients suffered from major complications during the transfer and vital signs and clinical features were similar between departure and on arrival. As transfer-related medical interventions were minor, these interventions could also be performed by trained paramedics according to predefined algorithms and may not necessarily require the presence of an accompanying physician. This protocol would be particularly helpful in networks where significant delays in initiating EVT can be avoided by not requesting a physician for transfer. Furthermore, removing experienced staff and equipment from rural areas may have a negative impact on the remaining population^12^.

Guidelines and recommendations for the inter-hospital transfer of critically-ill patients differ to a great extent and there is a lack of data in regards to staffing the transport units^22,23^. In our analysis, almost half of the patients finally did not receive EVT at our center after arrival and repeated imaging and this was due to extensive ischemic changes (i.e. ASPECTS < 6) in the majority of patients. It may be possible that faster transport without the need to call for an accompanying physician may have enabled EVT in some of these patients.

There is only little data available regarding the safety of inter-hospital transfer of stroke patients. One recent study has reported the frequency of transfer-related major (i.e. life-threatening) complications of 4.3% and minor complications of 22.6% among 253 patients. The presence of BAO, a NIHSS > 22 and a history of atrial fibrillation emerged as independent predictors of transfer-related complications in this study. However, the long travel distances (>150 km, median transfer time 92 minutes) and the involvement of only two stroke centers limit the overall generalizability to larger stroke networks^24^.

Almost 10% of our patients were intubated prior to transfer. Although this might reduce the door-to-groin-time at the EVT-centers as the patients do not have to be intubated before EVT, the continuous sedation and artificial ventilation carries several disadvantages: the patients´ neurological status cannot be monitored and the presence of an accompanying physician for transfer is mandatory, which might lead to even longer transfer times if the accompanying colleague is not readily available. Furthermore, the question if patients should be intubated for EVT is not yet fully answered, although there is evidence that a prolonged general anesthesia might be hazardous in patients undergoing EVT^25–27^. As expected, patients with BAO were intubated more often than patients with anterior circulation stroke. However, only 6.3% of the patients with BAO who were not intubated prior transfer required intravenous medication, suggesting clinical stability after initial assessment at the remote hospital.

Unsurprisingly, when comparing patients with and without medical intervention, the former had received IVT in a drip-and-ship approach more frequently, apparently due to a stricter management of blood pressure to avoid intracerebral hemorrhage^28,29^. It is moreover noteworthy that in our study population, patients with medical intervention were less likely to receive EVT and more likely to die at three months. Given the reasons why EVT was not performed in our study population, it seems implausible that the requirement for transfer-related medical interventions had a causal relationship but rather reflects an overall worse clinical status at baseline. An increased mortality and lower chance for successful EVT of stroke patients who are transferred for EVT has been described previously and was attributed to long transfer distances, arrival during off-hours and overall longer onset-to-treatment times^11,30,31^. Our analysis showed a significant association of intravenous thrombolysis, systolic blood pressure, heart rate and oxygen saturation at departure with intervention during transfer. However, it remains questionable if these parameters could be used to determine which patients need a physician as part of the transfer staff.

Our study has limitations. Although detailed data of vital signs and clinical status on departure and at arrival were available, we do not have continuous metrics and therefore cannot claim clinical stability during the whole transfer with the utmost certainty. The medication was given at the discretion of the accompanying staff and we must assume that medications were given according to guidelines. However, individual preferences of the transfer-physician may have triggered to apply specific medication and also not to apply specific medication during the transfer. As described earlier, an emergency physician is part of the helicopter crew and a physician routinely accompanies the patient during the ambulance-transport. However, we cannot exclude that the physicians at the remote hospitals staffed the ambulance with paramedics only in certain cases (e.g. short distance to the main hub, no accompanying physician timely available). We also included patients with basilar artery occlusion in our study due to their importance in tertiary stroke centers, although the evidence of benefit of EVT is lower in this group of patients^32,33^. Furthermore, we cannot comment on the proportion of EVT-eligible patients who were not transferred for evaluation of EVT. However, as we routinely transfer all EVT-eligible patients based on our network standard operation procedures we assume that this proportion is very low.

## Summary/Conclusions

In our telemedical stroke network, medical interventions during transfer for evaluation of EVT were required in a minority of patients with acute ischemic stroke and no patient suffered major complications associated with the transfer. It needs to be analyzed whether the presence of an accompanying physician during the transfer is associated with an improved functional outcome compared to transport with trained paramedics who act based on predefined treatment algorithms.

## Data Availability

The datasets generated during and/or analyzed during the current study are available from the corresponding author on reasonable request.
